# The cat is out of the bag: How parasites know their hosts

**DOI:** 10.1371/journal.pbio.3000446

**Published:** 2019-09-05

**Authors:** Elizabeth D. English, Boris Striepen

**Affiliations:** Department of Pathobiology, School of Veterinary Medicine, University of Pennsylvania, Philadelphia, Pennsylvania, United States of America

## Abstract

*Toxoplasma gondii* is a remarkably successful protozoan parasite that infects a third of the human population, along with most mammals and birds. However, the sexual portion of the parasite’s life cycle is narrowly limited to cats. How parasites distinguish between hosts has long been a mystery. A new study reveals that *Toxoplasma* identifies cats based on a single fatty acid, linoleic acid. Experimental manipulation of fatty acid metabolism by drug treatment turns a mouse into a cat in the “eye” of the parasite. This new model enables genetic crosses of an important human pathogen without the use of companion animals and opens the door to future discovery.

## Abstract 2

The parasite Toxoplasma gondii infects a wide range of mammals and birds but the sexual part of its life cycle only unfolds in cats. This Primer explores the implications of a new study that reveals the molecular cue by which the parasite distinguishes cats from other animals.

## Introduction

Any biology student who took a class in parasitology knows that parasites have life cycles of byzantine complexity, along which they are transmitted from one host to the next. In each host, they develop specific stages that, without fail, will have lengthy yet similar sounding Greek names that are truly painful to memorize. How parasites actually recognize and distinguish all these hosts is among the most fundamental questions in their biology, and one frequently asked by students. The unsatisfying answer given by those who lecture is typically that we do not know.

In a groundbreaking study published in this edition of *PLOS Biology*, Martorelli Di Genova and colleagues show that the parasite *Toxoplasma gondii* can tell a cat from a mouse by recognizing a single molecule, the fatty acid linoleic acid [1].

*Toxoplasma* is a small single-celled eukaryote that makes a living as a parasite of animals. In those animals, it invades cells and sets up a home within a specialized vacuole, where it multiplies rapidly [2]. Two days later, the host cell is filled with parasites that burst from their victim to infect new cells. A total of 30%–50% of the world’s human population are estimated to be infected with *Toxoplasma*. Fortunately, severe disease is relatively rare, despite the fact that so many of us carry this parasite for life. But when certain functions of the immune system are missing or flagging, as in HIV-AIDS patients or unborn children that are infected congenitally, *Toxoplasma* causes life-threating disease, most often in the form of encephalitis [3]. It thus appears that *Toxoplasma* is not a benign guest but that our immune system and the parasite evolved an uneasy but stable truce that shields us from damage and at the same time provides *Toxoplasma* lifelong residence [4].

**Fig 1:**
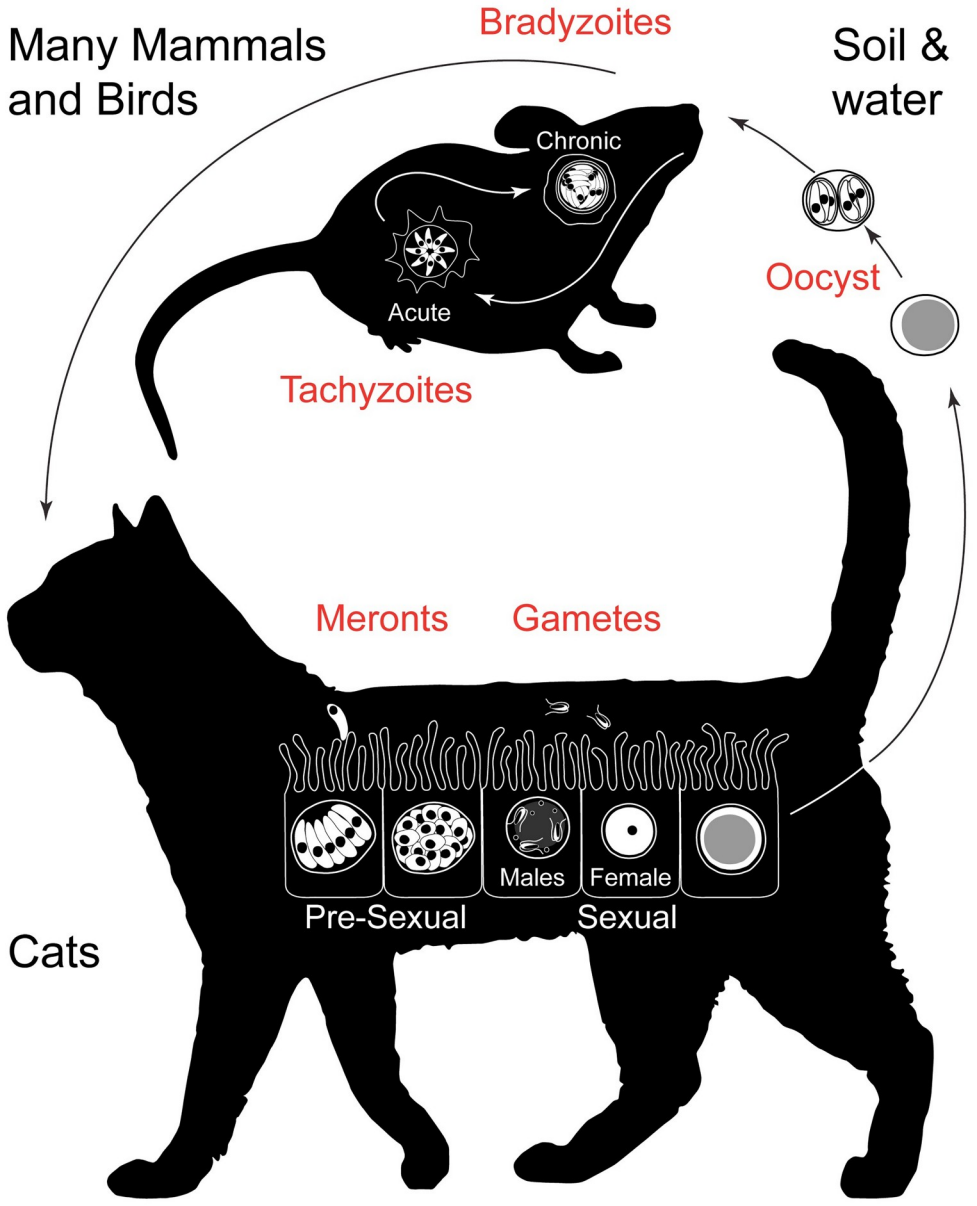
Simplified life cycle of *Toxoplasma gondii*. Many mammals and birds can serve as intermediate hosts of *T*. *gondii*. In these hosts, tachyzoites drive systemic infection. At some point, tachyzoites transform into cyst-forming bradyzoites that persist chronically for the lifetime of the host. When cats consume bradyzoite cysts, a different developmental pathway ensues. Several asexual replication cycles in the intestinal epithelium (merogony) are followed by differentiation into male and female gametes. Fertilization results in an immature oocyst that is shed with the feces into the environment. Meiosis and formation of infectious sporozoites occurs over the course of several days in the environment. Sporulated oocysts are highly infectious to intermediate and final hosts.

We are not the only host of this parasite. *Toxoplasma* is capable of infecting an astounding variety of mammals and birds, and within these animals, many tissues can harbor the parasite. However, the parasite’s sexual reproduction is restricted to cats, both domestic and wild members of the mammalian family Felidae, and plays out in their intestines (Fig 1). For the majority of its life cycle, *Toxoplasma* is haploid and reproduces asexually. The most commonly studied form of the parasite, the tachyzoite, replicates rapidly, disseminates throughout the host, and is responsible for disease. Within two weeks of infection, the host immune system will recognize, control, and kill tachyzoites. However, tachyzoites can differentiate into a slower-growing encysted form called bradyzoite [5]. These cysts are hidden in the brain and in muscle and escape immune detection and destruction in a fashion that is not fully understood. Tachyzoites and bradyzoites can be grown and studied in cell culture and mice, and driven by ever-improving genetic tools, we have learned much about their biology.

When an infected host is eaten by a carnivore or scavenger, its bradyzoites awaken from hibernation to take over the new host. In most hosts, they will revert to tachyzoites and disseminate through all tissues; however, should bradyzoites find themselves in the gut of a cat, they will follow a dramatically different playbook. Here, bradyzoites turn into merozoites, which solely infect intestinal epithelial cells, amplifying their numbers in a fashion that is distinct from their brethren, followed by differentiation into sexual parasites [6]. These come in two flavors: small male parasites (microgametes) that swim with the help of two flagella and find and fertilize female parasites (macrogametes) that remain within their host cell. The female then encases the resulting zygote in a sturdy shell, forming the oocyst. Immature oocysts are excreted with feces into the environment, where they undergo meiosis and the formation of eight new invasive stages to become infectious (Fig 1). They are environmentally hardy and contaminate drinking water or plants in large numbers.

**Fig 2:**
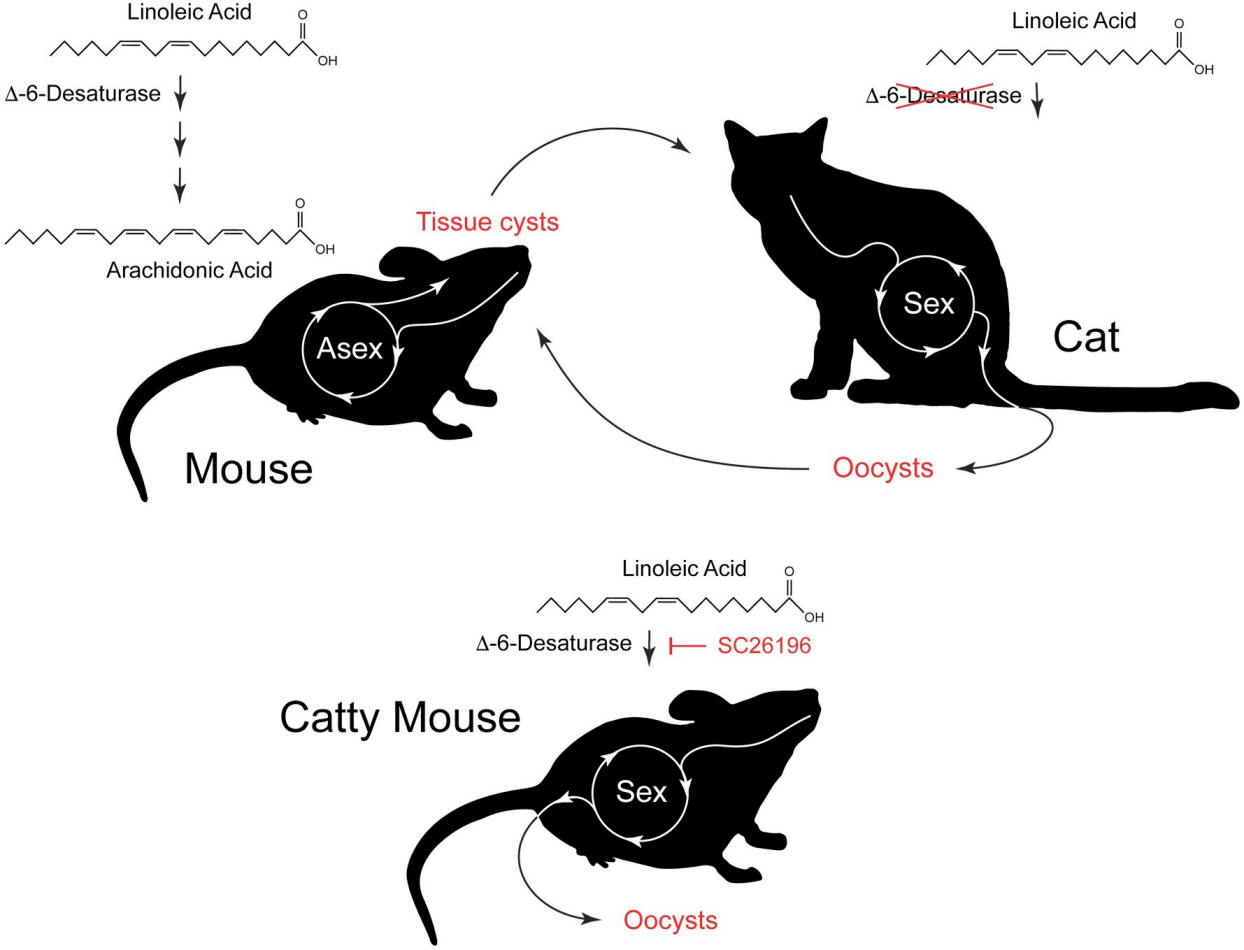
*Toxoplasma gondii* recognizes its final host, the cat, based on the abundance of the fatty acid linoleic acid. Most mammals, including mice, metabolize the linoleic acid they take up with their food into arachidonic acid. The enzyme Δ-6-desaturase is the committed step of this pathway. Cats lack this enzyme and thus accumulate linoleic acid to levels higher than other mammals. In the mouse, *T*. *gondii* follows an entirely asexual developmental path that leads to bradyzoite cysts in the brain of the mouse. When cats consume these cysts, a sexual program unfolds that leads to oocyst shedding with the feces. Martorelli Di Genova and colleagues [1] blocked Δ-6-desaturase in mice, leading to artificial accumulation of linoleic acid. Remarkably, when these mice are infected with *T*. *gondii*, they shed oocysts, identifying linoleic acid as the key to the recognition of the final host. SC26196, inhibitor of Δ-6-desaturase activity.

How does the bradyzoite know whether it arrived in the gut of a cat or a mouse? In this paper, Martorelli Di Genova and colleagues set out to develop a tissue culture system for *Toxoplasma* sex [1]. They established cat intestinal organoids (often referred to as “mini-guts”) and then infected them with bradyzoites. That produced some progress but was not enough to drive robust sexual development. *Toxoplasma* is known to scavenge many nutrients from the host; they thus next experimented with supplementing the culture media with different molecules, including lipids. On what must have been a great day in the laboratory, they discovered that supplementation with linoleic acid, but not oleic acid, promoted progression into merozoites and eventually macro- and microgametes. This correlates nicely with the fact that linoleic acid is the dominant fatty acid in the serum of cats. Most mammals rapidly turn linoleic acid over to arachidonic acid. Cats lack Δ-6-desaturase, the enzyme catalyzing the first committed step for this conversion, and have to take arachidonic acid from their food [7]. Linoleic acid thus sticks around and accumulates. Armed with this information, the authors hypothesized that the parasites sense linoleic acid. To put this to a rigorous test, they treated infected mice with an inhibitor of Δ-6-desaturase, and for good measure provided them with chow rich in linoleic acid. Implementing this simple nutritional regime resulted in mice that showed meronts and gametes in their intestines and that shed oocysts in their feces (Fig 2). Further tests demonstrated that these were infectious and in any way “as good as cat.”

This discovery is of tremendous practical value to advance research in *Toxoplasma* but also in two related parasites that cause malaria and diarrhea (*Plasmodium* and *Cryptosporidium*). The main benefit of the new model is that it allows classical genetic crosses between different parasite strains to identify the genes behind their biological differences. Importantly, this does not require any prior knowledge of the mechanism. A famous example for this approach comes from the malaria field. Chloroquine, a stalwart drug that saved millions of lives, had lost its value due to rampant parasite drug resistance. Researchers crossed a drug-sensitive parent parasite with a resistant parasite and then analyzed the “children,” which carried different mixes of their parents’ genomes. Comparing the genomes of sensitive and resistant progeny led to a mutated transport protein that caused resistance [8]. Similarly, studies in *Toxoplasma* that crossed benign and highly virulent parasites led other researchers to discover how mice protect themselves from infection and how more aggressive “killer” parasites can outsmart that protection [9]. The malaria studies required the use of mosquitoes and chimpanzees and the *Toxoplasma* studies, cats. The National Institutes of Health (NIH) halted the use of chimps in 2015 [10], and the United States Department of Agriculture (USDA) this year shuttered a laboratory that for decades specialized in cat experiments with *Toxoplasma*. The highly respected director of the lab, Dr. Dubey, had discovered the *Toxoplasma* life cycle in the 1970s and is a coauthor of the current study. USDA’s decision to abandon this work sparked widespread protest in the infectious disease research community [11]. The new laboratory mouse model thus could not be more timely. Using mouse experiments to conduct crosses will undoubtedly lead to a wave of exciting discoveries.

One of the things that remain to be worked out is the molecular mechanism by which *Toxoplasma* senses differences in the abundance of linoleic acid. The authors favor a model that implies signaling rather than nutrition. That appears reasonable: polyunsaturated fatty acids like arachidonic and linoleic acid are well known second messengers with important roles in a wide variety of biological phenomena. The fact that the parasite responds to the linoleic acid cue in tissue culture and that the response is unlikely to be essential for the growth of asexual parasites suggest that genetic screens targeting the mechanism should be feasible. Full genome clustered regularly interspaced short palindromic repeats/CRISPR-associated protein 9 (CRISPR/Cas9) knock-out screens are feasible in *Toxoplasma* [12] and could lead the authors to the responsible parasite genes.

While the authors were able to observe merozoites and gametes in cultured cells, cultures did not efficiently produce oocysts, the meiotic spores that are the final product of parasite sex. That suggested to them that a yet unknown physiological requirement for sex is met in whole animals, but not in culture. A variety of problems in the parasite’s romantic life are conceivable: imperfect development of gametes, lack of fertilization, or lack of meiosis and oocyst formation. This is remarkably similar to observations made for the related apicomplexan parasite *Cryptosporidium*, a leading global cause of diarrheal disease in infants. This parasite does have sex in mice, but tissue culture falls similarly short. Recent studies on this organism suggest that gametes form fine in culture and that males are capable of finding females; however, fertilization does not happen [13]. This is improved by using more complex organoid culture models, in which the formation of infectious oocysts can be observed [14,15], albeit not at the full efficiency of an animal model. Whatever physiology is missing in transformed cell lines thus appears present in organoids, but still not quite to the level provided by a whole animal. Cracking this riddle could allow genetic crossing in culture without any animal use, which could be scaled tremendously. This would open the way to truly powerful forward genetics to understand and ultimately defeat parasites that cause important diseases in humans and animals. The discovery reported in the current paper brought the field an important step closer to this goal and is one of only a handful of molecular mechanisms that explain parasite host specificity.
